# P-1637. Limitations of Upper Respiratory Tract Testing in Diagnosing COVID-19 Pneumonia: A Retrospective Cohort Study

**DOI:** 10.1093/ofid/ofaf695.1813

**Published:** 2026-01-11

**Authors:** Ming-Wei Lin, Yun-Ting Chung, Shian-Sen Shie, Po-Yen Huang, Ching-Tai Huang

**Affiliations:** Chang Gung University, Taoyuan, Taoyuan, Taiwan (Republic of China); Chang gung university, Taoyuan, Taoyuan, Taiwan; Chang Gung Memorial Hospital, Taoyuan, Taoyuan, Taiwan; Chang Gung Memorial Hospital, Taoyuan, Taoyuan, Taiwan; Chang Gung Memorial Hospital, Taoyuan, Taoyuan, Taiwan

## Abstract

**Background:**

Upper respiratory tract (URT) testing including rapid antigen tests and RT-PCR of nasopharyngeal and oropharyngeal swabs has been the cornerstone for diagnosing SARS-CoV2 infection. However, emerging evidence suggests that negative URT results may not definitively exclude COVID-19 pneumonia, potentially delaying appropriate treatment. This study aims to evaluate the diagnostic yield of lower respiratory tract (LRT) testing and its implications for clinical severity.

**Figure:**
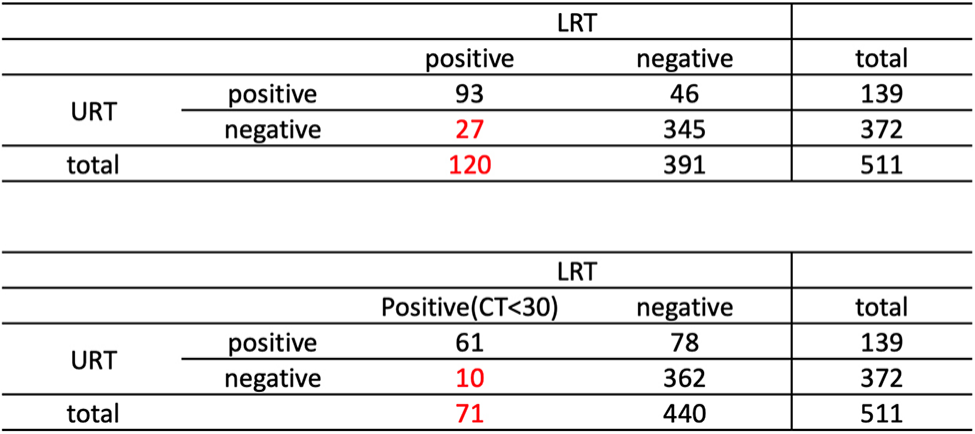
Concordance Between URT and LRT SARS-CoV-2 Testing Results Tables showing the diagnostic concordance between upper respiratory tract (URT) and lower respiratory tract (LRT) SARS-CoV-2 tests in 511 patients. The first table includes all LRT-positive cases; the second applies a stricter LRT positivity definition with CT<30. A notable proportion of LRT-positive patients had negative URT results in both scenarios.

**Figure:**
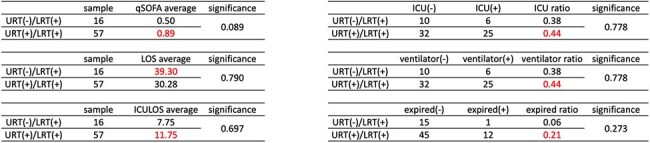
Clinical Severity Comparison Between URT(-)/LRT(+) and URT(+)/LRT(+) Groups Comparison of clinical severity in measured outcomes between URT(-)/LRT(+) and URT(+)/LRT(+) groups. Outcomes assessed included qSOFA score, hospital length of stay (LOS), ICU admission rate and duration (ICULOS), ventilator usage, and mortality rate. No statistically significant differences were observed in any of the measured outcomes between the two groups.

**Methods:**

We conducted a retrospective analysis of 511 cases who underwent simultaneous upper respiratory tract and lower respiratory tract SARS-CoV2 testing between January 2020 and May 2024. URT specimens were obtained via nasopharyngeal or oropharyngeal swabs, while LRT specimens included sputum, endotracheal aspirates, or bronchoalveolar lavage fluid. The primary outcome was the proportion of URT(-) results among LRT(+) cases. A secondary analysis compared clinical severity between URT(-)/LRT(+) and URT(+)/LRT(+) groups, assessing qSOFA scores, hospital length of stay, ICU admission rates, duration of ICU stay, ventilator usage, and mortality rates.

**Results:**

Among the 120 LRT(+) cases, 22.5% (27/120) had negative URT results. When defining LRT positivity as a cycle threshold (Ct) value < 30, the proportion of URT(-) cases was 14.08% (10/71). In the subset of 73 patients evaluated for clinical severity, no significant differences were observed between URT(-)/LRT(+) and URT(+)/LRT(+) groups across all measured outcomes, including qSOFA scores (0.50 vs. 0.89, P=0.089), hospital length of stay (39.30 vs. 30.28 days, P=0.790), ICU stay duration (7.75 vs. 11.75 days, P=0.697), ICU admission rates (38% vs. 44%, P=0.778), ventilator usage (38% vs. 44%, P=0.778) and mortality rates (6% vs. 26%, P=0.273).

**Conclusion:**

Our findings indicate that reliance on URT testing may miss approximately 14–22% of COVID-19 pneumonia cases, underscoring the importance of incorporating LRT testing into diagnostic protocols, especially for patients with high clinical suspicion. The absence of significant differences in clinical severity between URT(-)/LRT(+) and URT(+)/LRT(+) groups suggests that disease progression may be independent of URT viral load, highlighting the need for comprehensive diagnostic strategies.

**Disclosures:**

All Authors: No reported disclosures

